# Supplementary material for: The P2Y2 Receptor C-Terminal Tail Modulates but Is Dispensable for β-Arrestin Recruitment
Source: Int J Mol Sci. 2022 Mar 22;23(7):3460. doi: 10.3390/ijms23073460 (PMC8999042; doi:10.3390/ijms23073460)
Supplement: Supplementary file 1 [file ijms-23-03460-s001.zip › ijms-1622926-supplementary.pdf]

The P2Y$_2$ receptor C-terminal tail modulates but is dispensable for β-arrestin recruitment

Eline Pottie, Jolien Storme, Christophe P. Stove*

Laboratory of Toxicology, Department of Bioanalysis, Faculty of Pharmaceutical Sciences, Ghent University, Campus Heymans, Ottergemsesteenweg 460, B-9000 Ghent, Belgium.

* Corresponding Author: Christophe Stove, Laboratory of Toxicology, Department of Bioanalysis, Faculty of Pharmaceutical Sciences, Ghent University, Ottergemsesteenweg 460, 9000 Ghent, Belgium, Phone: +32 9 264 81 35, Fax: +32 9 264 81 83, E-mail: Christophe.Stove@UGent.be

Supplementary Materials

Table S1	PCR primers, restriction enzymes, and experimental conditions for molecular cloning
Figure S1	Time-luminescence profiles obtained with ATP

Supplementary Table S1: PCR conditions (a-c) and restriction enzymes (RE: d) used for the development of NanoBiT® fusion constructs. A: primers (5' – 3') containing the specific restriction site (underlined); b: annealing temperature; c: extension time.

Template sequence		Primers (F: forward – R: reverse)[a]	Tm (°C)[b]	Ext. time (s)[c]	RE[d]	Fusion construct
βarr1 NanoBiT® plasmid construct development						
βarr1	F	ATCCAAGAGCTCAGATGGGCGACAAAGGG	66.0	40	SacI	SmBiT-βarr1
	R	ATCCAAGAGCTCTCATCTGTTGTTGAGC				
$P2Y_2R$ NanoBiT® plasmid construct development						
$P2Y_2R$	F	ACTCAACTCGAGACCATGGCAGCAGACC	71.9	30	XhoI	$P2Y_2R$-LgBiT and $P2Y_2R$-SmBiT
	R	ACTCAACTCGAGCCCAGCCGAATGTCC				

Template sequence		Phosphorylated primers	Tm (°C)[b]	Ext. time (min: sec)[c]	Fusion construct
Insertion HA-tag					
$P2Y_2R$-LgBiT	F	TACCCATACGATGTTCCAGATTACGCTATGGC AGCAGACCTGG	72	1:50	(2xHA)-$P2Y_2R$-LgBiT
	R	AGCGTAATCTGGAACATCGTATGGGTACATG GTCTCGAGCCAGAATTCC			
Adjustment of the linker sequence					
$P2Y_2R$-LgBit	F	GGTGGAGGTGGTGTCTTCACAC	67.6	1:40	$P2Y_2R$**L**-LgBiT
	R	CAGCCGAATGTCCTTAGTGTTCTCG			
(2xHA)-$P2Y_2R$-LgBit	F	GGTGGAGGTGGTGTCTTCACAC	67.6	1:40	(2xHA)-$P2Y_2R$**L**-LgBiT
	R	CAGCCGAATGTCCTTAGTGTTCTCG			
Truncation of the C-terminus					
$P2Y_2R$-LgBit	F	GGCTCGAGCGGTGGTGGCG	67.5	1:35	$P2Y_2R$**Δ322**-LgBiT
	R	TGGCTTGGCATCTCGGGCAAAGC			
$P2Y_2R$-LgBiT	F	GGTGGAGGTGGTGTCTTCACAC	68.3	1:35	$P2Y_2R$**LΔ322**-LgBiT
	R	TGGCTTGGCATCTCGGGCAAAGC			
(2xHA)-$P2Y_2R$-LgBit	F	GGCTCGAGCGGTGGTGGCG	67.5	1:35	(2xHA)-$P2Y_2R$ **Δ322**-LgBiT
	R	TGGCTTGGCATCTCGGGCAAAGC			
(2xHA)-$P2Y_2R$-LgBit	F	GGTGGAGGTGGTGTCTTCACAC	67.5	1:35	(2xHA)-$P2Y_2R$**LΔ322**-LgBiT
	R	TGGCTTGGCATCTCGGGCAAAGC			
Mutation of phosphorylation sites in IL3					
$P2Y_2R$**LΔ322**-LgBiT	F	TAGGGCCAAGCGCAAGGCCGTGCGC	72	1:37	$P2Y_2R$**LΔ322**-T232**A**-S233**A**-S243**A**-LgBiT
	R	GGCAGGCCGCCCGCGGCCCCGTAGG			
(2xHA)-$P2Y_2R$**LΔ322**-LgBiT	F	TAGGGCCAAGCGCAAGGCCGTGCGC	72	1:37	(2xHA)-$P2Y_2R$**LΔ322**-T232**A**-S233**A**-S243**A**-LgBiT
	R	GGCAGGCCGCCCGCGGCCCCGTAGG			

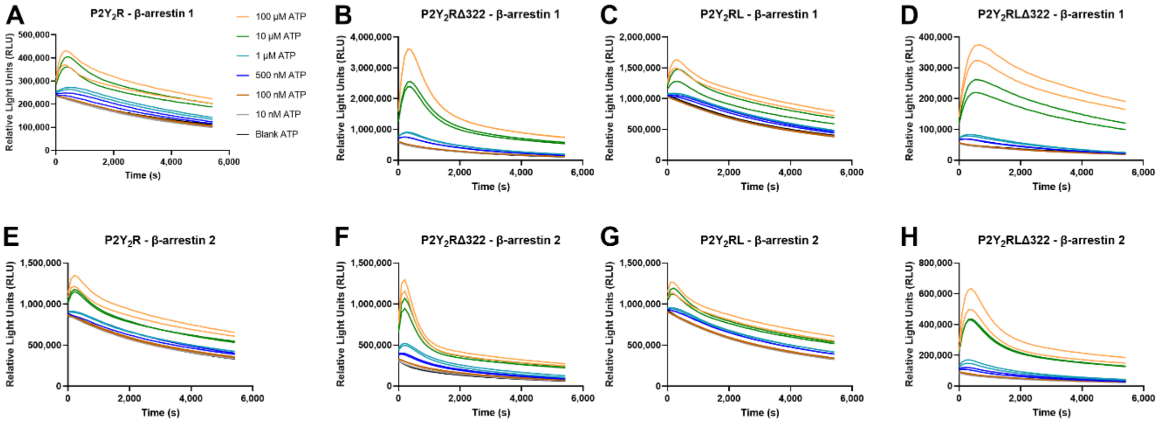

Supplementary Figure S1: Time-luminescence profiles obtained in the NanoBiT® assay by recruitment of βarr1 (panels A - D) or βarr2 (panels E - H) by the full length $P2Y_2R$-LgBiT (panels A and E), the C-terminally truncated $P2Y_2R$**Δ322**-LgBiT construct (B and F), $P2Y_2R$**L**-LgBiT (C and G) or $P2Y_2R$**LΔ322**-LgBiT, induced by endogenous agonist ATP. Data are from one representative experiment, out of three independent experiments.
